# When Parasitoid Males Make Decisions: Information Used when Foraging for Females

**DOI:** 10.1371/journal.pone.0046706

**Published:** 2012-10-03

**Authors:** Claire M-S Dufour, Philippe Louâpre, Joan van Baaren, Véronique Martel

**Affiliations:** Université de Rennes I, Unité Mixte de Recherche (UMR) Centre National de la Recherche Scientifique (CNRS) 6553 EcoBio, Rennes, France; University of Bristol, United Kingdom

## Abstract

Optimal foraging models predict how an organism allocates its time and energy while foraging for aggregated resources. These models have been successfully applied to organisms such as predators looking for prey, female parasitoids looking for hosts, or herbivorous searching for food. In this study, information use and patch time allocation were investigated using male parasitoids looking for mates. The influence of the former presence of females in absence of mates and the occurrence of mating and other reproductive behaviours on the patch leaving tendency was investigated for the larval parasitoid *Asobara tabida*. Although males do not modify their patch residence time based on the number of females that visited the patch, they do show an increase in the patch residence time after mating a virgin female and performing courtship behaviour such as opening their wings. These results are in concordance with an incremental mechanism, as it has been described for females of the same species while foraging for hosts. The similarities between males and females of the same species, and the conditions under which such a patch-leaving decision rule is fitted are discussed. This is the first study describing an incremental effect of mating on patch residence time in males, thus suggesting that similar information use are probably driving different organisms foraging for resource, regardless of its nature.

## Introduction

Optimal foraging models predict how organisms should behave in order to maximise their lifetime fitness gain when foraging for resources (e.g. food, hosts or sexual partners) [1], [2]. When facing a resource of variable quality aggregated in patches of different sizes, organisms are expected to adjust their residence time to the patch quality to maximise their resource intake rate as predicted by Charnov's Marginal Value Theorem [3]: the higher the patch quality, the longer the residence time. This adaptive foraging behaviour was observed in a wide range of taxa such as insects, birds, rodents, cattle or humans [4]–[8].

Several authors attempted to explain patch time allocation of parasitoids or predators using different patch-departure decision rules (the so-called proximate mechanisms) such as giving-up time, giving-up density, motivation, time expectation rule, etc. [9]–[15]. McNamara and Houston [16] pointed out recently the importance of studying the evolution of these mechanisms in relation with the complexity of the environment and state that behavioural ecologists have to consider the internal state of the forager in order to understand how evolution and environment shaped the behaviour. Some of these patch-departure decision rules are based on the motivation (or responsiveness) to stay in the patch. Motivation is defined as a variable energizing, directing and selecting a given action [17]–[20]. Even if it is a hidden and thus not directly observable variable [21], a variety of animals (e.g. parasitoids, bumblebees, human) with different cognitive abilities were shown to make their decision to leave a patch in manners consistent with motivation models (e.g. [7], [22]).

**Figure 1:**
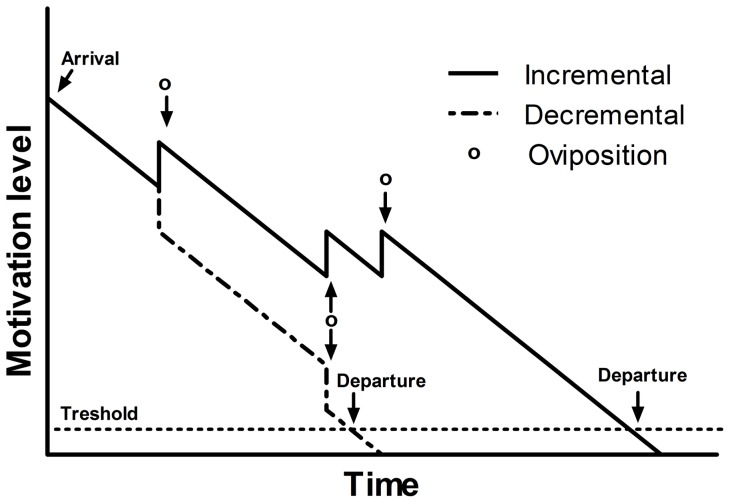
The main motivation-based mechanisms of decision-making described for female parasitoids. In the incremental mechanism, motivation level increases with an oviposition while in the decremental mechanism, motivation level decreases with an oviposition, leading to a shorter patch residence time with the latter. In both cases, the organism leaves the patch when its motivation level reaches a threshold.

Models of patch departure assume that sensorial cues must be perceived during foraging [12], [23], [24]. The tendency to stay at arrival in the patch thus depends on the estimated patch quality. In the absence of any resource encountering event (see explanation below), the tendency to stay is assumed to decrease steadily with time until a threshold is reached; the patch is then abandoned [12] (Figure 1). As a result, as long as the decrease in the tendency to stay (or motivation) is independent of patch quality, patch residence time depends on the estimated patch quality: in many predatory mites, chemicals emitted by the prey inform predators about the patch quality and they increase their residence time accordingly [25], [26]. The tendency to stay during foraging also depends on different intra-patch factors such as the resource encountering. Waage [12] suggested that the motivation rises suddenly when the ichneumon *Venturia canescens* lays an egg in a healthy host, thus defining an incremental motivation model of patch departure (Figure 1). This incremental model was suggested in several parasitoid species (reviewed by [7]), bumblebees [27] and humans [22]. In some cases, encountering the resource decreases instead of increasing the tendency to stay: such a decremental mechanism (Figure 1) has been suspected in a number of insect parasitoids (reviewed by [7], [28]) and bumblebees [29].

The use of an incremental or a decremental mechanism depends on several factors such as the quality of the resource (increment/decrement if the resource is of high/poor quality) [24], the resource distribution among patches (increment/decrement when clumped/uniform) [30], and the cues reliability of the patch quality at arrival (increment/decrement when the forager has unreliable/reliable information) [31]. A switch between both mechanisms has even been observed depending on the experience of the forager in *Aphidius rhopalosiphi* [32].

Both the patch-leaving decision rules and the information use by foragers have been extensively studied in insect parasitoids, mainly in females searching for hosts in which to lay their eggs (but see [33], [34]). However, in many species such as gregarious or quasi-gregarious insect parasitoid (e.g. [34]), males, foraging for females to mate with, just like females foraging for hosts to parasitize, exploit patches of resources, and this foraging behaviour should thus have important effect on the male's fitness. As males' fitness is maximized while maximizing the number of mates [35], males should optimize their time allocation to maximize their number of mates [34] if (i) they are time-limited (and not sperm-limited), (ii) females occurs in patches, which is likely to happen on either emergence or foraging patches in many parasitoid species, especially the gregarious or quasi-gregarious ones with synchronized emergence and local mating, and (iii) the fitness gain on a patch decreases with time. Fitness gain is expected to decrease with patch exploitation as in insects, one mating is normally sufficient to fill the female's spermatheca [36]–[37]. Little is known however about the information used by males underlying the decision to leave a patch of females. Martel *et al*. [34] recently showed that the number of virgin females present as well as the number of encounters with parasitized hosts influence the patch residence time of males of a minute egg parasitoid (*Trichogramma euproctidis*); however, in that study the number of females on the patch was not controlled during the observation and the decision process is still unknown.

The main objective of this study is to understand which factors or information, in male parasitoids, influence the decision to leave a patch of females. We hypothesize that (i) males adjust their patch residence time (and their behaviours) with the perceived patch quality at arrival, staying longer in patches of higher perceived quality, and that (ii) courtship and mating events influence the decision of males to leave the patch. The studied species is the *Drosophila* larval parasitoid, *Asobara tabida* (Hymenoptera, Braconidae).

## Materials and Methods

### Insects

This study does not include any field work. The *Asobara tabida* strain used in this study originated from Avignon, France and was collected using banana baited traps in 2009; the insects were collected at a research station in Avignon and permits were obtained when necessary. The rearing was started using approximately 50 females and 20 males. The parasitoid was reared on *Drosophila subobscura* (collected in the Netherlands at the end of the 1980 decade) in glass jars (Ø 4.5 cm) containing a substrate of agar-nipagine (the agar serves as a substrate and the nipagine is an anti-fungi) topped with a thin layer of yeast *Saccharomyces cerevisiae* (Saccharomycedacae) (produced by Fermipan ®); the yeast serves as food for the *Drosophila* larvae. The cultures of both hosts and parasitoids were maintained in a climate room (20°C, 60% RH, and LD 16∶8 h).

### General Biology

*Asobara tabida* is a larval parasitoid of *Drosophila* species: females lay their eggs preferentially in late-first to early-second instar larvae and one adult emerges from the host pupa [38]. *Drosophila* parasitoids are protandrous, i.e. males emerge before females [39]. Soon after emergence, mating is expected to occur mainly locally, leading to frequent inbreeding [39]. The typical male mating behaviour in *Drosophila* parasitoids, as for parasitic wasps in general, is as follow: attraction, recognition, orientation, wing vibration (or wing fanning), antennation, head movement, leg tapping, copulation and post-copulatory grooming [39]–[40]. Wing fanning is the most conspicuous element of mating behaviour in these insects [40].

Once mated, females will search for hosts for oviposition. Their habitat is mainly fermented fruits or sap fluxes containing different numbers of host larvae aggregately distributed [41]. Females respond to chemical cues related to hosts [42]–[43] and to vibrations [38], and adjust their patch residence time depending on oviposition events [43], perceived patch quality [44] and experience [44]–[45]. Females are likely to be aggregated like their hosts – even if the former are more mobile than hosts – either in their emergence patch or on their foraging patches [43], although males are not attracted to oviposition sites [46]. Males have mature sperm at emergence (V. Martel, personal observation), but the possible production during their adult life is not known.

### General method

Two days before emergence, parasitised pupae from the culture were isolated in gelatin capsules (0.68 mL) to obtain virgin individuals of known age. Males used in all experiments were 24 h old (except when otherwise mentioned), virgin, fed with a diluted honey solution and placed in a clean Petri dish (Ø 8.5 cm) containing a thin agar-nipagine layer the day of their emergence. All females used were virgin, 3 to 6 days old, fed and kept in pots (Ø 4.5 cm) containing a substrate of agar-nipagine.

In every experiment, a light table was used to maintain the individuals on the bottom of the Petri dish by phototaxis. All experiments were video-taped and analysed using the computer software package The Observer v3.0 [47].

#### 1. Preliminary experiment: Male mate choice

In the experiments evaluating patch residence time (see below), only virgin females have been used. However, once the male mated one female, he was in presence of a mated female. Because of that, mate choice tests were performed first to evaluate if males would preferentially mate females depending on their mating status, suggesting either discriminative capacities or rejection by the female. One 1–4 d old virgin male was introduced in a Petri dish (Ø 8.5 cm) containing two 3–5 d old females (one virgin and one mated). Mated females were obtained 30 minutes before the beginning of the experiment by putting one virgin female with one virgin male until copulation was observed. Observations started as soon as the male was introduced in the Petri dish and ended after the first mating, or after 30 minutes if no mating occurred (only one out of 16 observations). The following events were recorded: first courtship and first mating.

#### 2. Influence of the former presence of females on patch residence time

To determine if the chemicals deposited by females that previously visited the patch (a potential proxy of patch quality) influences male patch residence time, patches that have been visited by females were created. One or 10 virgin females were placed in the centre of a Petri dish (Ø 8.5 cm) containing a thin layer of agar-nipagine, under the lid of a smaller Petri dish (Ø 4 cm) delimiting the patch. After 30 minutes, the females and the lid of the small Petri dish were removed and a male was immediately introduced into the large Petri dish (Ø 8.5 cm), but outside the patch (n = 15 males for each treatment). A similar experiment was conducted, but on clean patches on which no female has walked (n = 24), as a control to determine if males perceive the former presence of females. Observations started as soon as the male walked inside the patch and ended when he left it for at least 60 s or when he landed on the Petri dish cover or sides. The following events were recorded: entering the patch, leaving the patch, wing opening and wing fanning (typical courtship behaviours). Males open their wings quite frequently when in presence of virgin females, but not so frequently in front of a mated one (V. Martel, personal observations).

#### 3. Influence of mating on patch residence time

**Figure 2:**
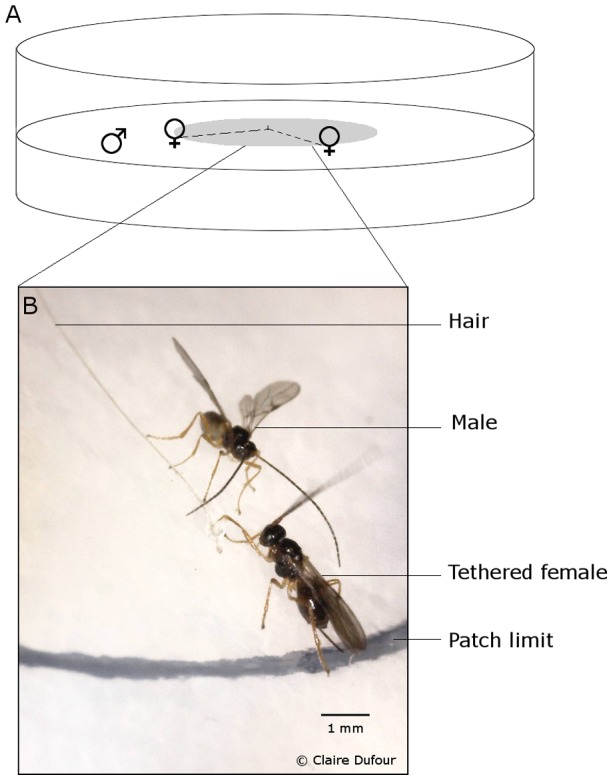
The experimental set-up used to determine the impact of mating on males' patch residence time. **A**. Two virgin females are attached by the leg to alcohol-washed hair (dashed line) on a delimited patch (grey area). **B**. A picture of a free male approaching a virgin tethered female in a patch.

To evaluate the impact of mating and courtships events on male patch residence time, a patch of females (*i.e*. an area where the male could freely enter, mate and leave without the female escaping) has been created (Figure 2). A Petri dish (Ø 8.5 cm) containing a thin layer of agar-nipagine in the centre of which two alcohol-rinsed hair were inserted before the substrate solidified was used. The other end of the hair was glued (glue SADER max) to the fore right leg of an anesthetised virgin female (Video S1), delimiting a patch of two tethered females whose radius is the hair length (Ø 4 cm). Hence, females can move freely within the patch area. The use of tethered females allows keeping the number of females present on the patch constant during the whole observation (in opposition to the previous study on male patch time allocation [34]). Each patch was previously visited by five virgin females (same procedure as in experiment 2) in order to increase the attractiveness of the patch for the tested males, and consequently decreases the frequency of males not visiting the patch. Although the female behaviour might be altered by the experimental procedure [48]–[49], preliminary observations comparing males in a Petri dish with a tethered female, or with a free female showed similar males' reproductive behaviours (C. Dufour, personal observations).

Observations started as soon as the male entered the patch and ended when the male left the patch for more than 60 s or landed on the cover or sides of the Petri dish (n = 16). The following events were recorded: entering the patch, leaving the patch, wing opening, wing fanning, mating and female escape.

### Statistical analyses

All statistical analyses were performed using R 2.12.0 software [50].

In the male mate choice experiment, the ratio of virgin females and already mated females courted or mated were compared to a 50∶50 probability using χ^2^ tests.

The proportion of males entering the patch previously visited by females was compared to the proportion of males entering the clean patch using a contingency table followed by a χ^2^ test. To estimate the effect of both the number of females that visited the patch and mating on the patch residence time, Cox proportional hazards models were used [51], [52]. The following covariates were used when applicable: number of females that previously visited the patch, mating with a virgin female, re-mating a female, female escape, wing opening, wing fanning and temporary exit. The parameter “female encounter” was not tenable, as in such a small arena, both individuals are almost always in proximity. In addition, the female to which the behaviours “wing opening” or “wing fanning” was addressed was not discriminated as it was not always clear. A thorough description of this model can be found in the literature dealing with patch-leaving decision rules [53]. The proportional hazards model expresses the data in terms of patch-leaving tendency (*i.e*. hazard rate), which is the probability per unit of time that a male leaves the patch, given that he is still on it. A hazard ratio above one indicates an increase in the male's patch-leaving tendency, while a hazard ratio below one shows a decrease in the patch-leaving tendency.

## Results

### 1. Preliminary experiment: Male mate choice

When given the choice, males prefer to court (χ^2^ = 7.14, d.f.  = 1, P = 0.008) and to mate (χ^2^ = 15, d.f.  = 1, P<0.001) virgin females.

### 2. Influence of the former presence of females on patch residence time

Fourteen out of 24 males did not enter the clean patch, which is significantly higher than in the patch previously visited by females, where only 6 out of 36 males did not visit the patch (χ^2^ = 11.25, d.f.  = 1, P = 0.002).

Neither the number of females that previously visited the patch, nor any of the tested events significantly influenced the patch-leaving tendency for males (Table 1); the time spent on the patch (mean patch residence time ± SD: patches visited by one female, 127.95±185.83 s; patches visited by 10 females, 157.09±212.19 s) is thus independent of the number of females that previously visited the patch and the performed behaviour in absence of females.

**Table 1:** Estimated regression coefficients (*β*) and hazard ratios (exp (*β*)) for covariates of the Cox model on the patch-leaving tendency of males *A. tabida* in a patch previously visited by females.

	*β*	exp (*β*)	*z*	*P*-value
Number of females that visited the patch	−0.0284	0.972	−0.598	0.550
Wings opening	0.526	1.692	0.807	0.420
Wing fanning	−0.125	0.883	−1.485	0.137
Temporary exit	0.395	1.484	1.934	0.053

### 3. Influence of mating on patch residence time

While mating with a virgin female significantly decreased the patch leaving tendency by a factor of 3.959, re-mating an already mated female had no effect on the patch leaving tendency (mean patch residence time ± SD: 1095.54±937.88 s) (Table 2). Opening their wings also decreased the patch leaving tendency, while temporary exits from the patch increased it. No other covariates had a significant effect.

**Table 2:** Estimated regression coefficients (*β*) and hazard ratios (exp (*β*)) for covariates of the Cox model on the patch-leaving tendency of males *A. tabida* in a patch of females.

	*β*	exp (*β*)	*z*	*P*-value
Mating with a virgin female	−3.959	0.019	−2.837	0.005
Re-mating a female	0.266	1.305	1.255	0.209
Wings opening	−0.434	0.648	−2.657	0.008
Wing fanning	0.080	1.083	1.293	0.196
Female escape	−0.146	0.864	−1.525	0.127
Temporary exit	1.656	5.237	3.172	0.002

**Figure 3:**
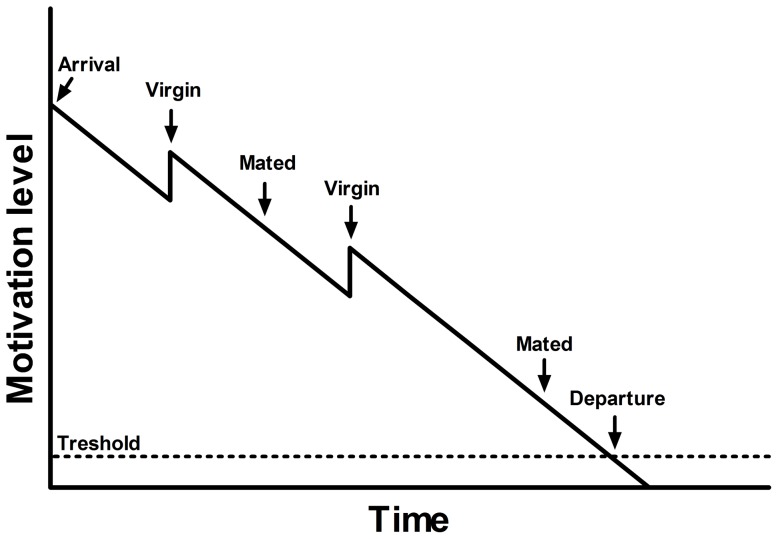
The suggested proximate mechanism underlying decision-making in male *A. tabida*. While mating a virgin female increases the tendency to stay on the patch, consequently increasing patch residence time, re-mating an already mated female has no impact on the residence time.

## Discussion

Our results show that male *A. tabida* use different information sources in their exploitation of a patch of resource, just like females do. Their residence time in a patch of females depends on the number of mating with virgin females and courtship behaviour such as wings opening, but not on the number of mating with already mated ones, nor on the number of females that previously visited the patch. The type of information used by male parasitoids for patch departure decision (i.e. the resource, mates) and its impact on patch residence time is thus similar to the one used by females of this species (i.e. the resource, hosts) even though some differences will be discussed.

Male *A. tabida* visit patches that have been previously visited by females significantly more frequently than clean patches, thus indicating that females lay a trail of a substance, which could be a sex pheromone, perceived by males. Sex pheromones are commonly used by insect males to locate and choose females [54]. After perceiving such infochemicals emitted by virgin females, males of parasitoid species such as *Trichogramma brassicae* and *Aphelinus asychis* tend to stay on the substrate, even in the absence of females [55].

Although male *A. tabida* do respond to the former presence of females by entering the patch, the decision of when to leave a patch previously visited by females did not depend on the number of females which visited it. In female parasitoids, infochemicals such as kairomones emitted by hosts were shown to be a highly detectable and reliable proxy of patch quality [28]. For example, patch residence of female *A. tabida* in patches containing host kairomones (but no hosts) depends on their amount: the higher the kairomone level, the longer the residence time [42], [44]. In the present study, males' lack of response to the number of females that previously visited the patch could be explained by the saturation of the patch medium absorption capacity independently of the number of virgin females present, or on the incapacity of males to evaluate the number of females that previously visited the patch. As females *A. tabida* are more mobile than hosts (i.e. *Drosophila* larvae), using chemicals emitted by females, potentially a sex pheromone deposited on the substrate might be less reliable for males than hosts' kairomones: females might have already flown away when males arrive on the patch. Other cues such as mating or encounters might then be more reliable. In addition, males' tendency to stay in the patch, even if independent of the number of females that previously visited it, could depend on other physiological factors (such as the male's age, nutritional status, or mating status) or on inter-patch travel time as observed in females (reviewed by [7]). Finally, the use of visual cues, tactile cues, or cues related to hosts in combination to cues related to females – as it has been shown to be attractive for males in other parasitoids species [56] – would be interesting to test, even though males were not shown to be attracted to oviposition sites [46].

In male *A. tabida*, each mating with a virgin female decreases the patch-leaving tendency, consequently increasing patch residence time on the patch of females. Both male and female *A. tabida* are thus expected to similarly use information in their patch-leaving decision, in concordance with an incremental mechanism (see Figure 1), as females seem to be distributed like hosts. The tendency of the male to stay on the patch of females thus seems to depend on the number of virgin females inseminated: the initial tendency to stay probably decreases with time and suddenly increases each time the male mates with a virgin female (Figure 3). Such an incremental mechanism was found in females of many parasitoid species [7]. However, mating with already mated females did not modify the male's decision to leave the patch, in concordance with our choice tests where male *A. tabida* discriminate females according to their mating status (even if in the choice test, the mated female was inseminated by another male). As in insects, one mating is normally sufficient to fill the female's spermatheca [36], [37], fitness is thus maximized when a male inseminate a virgin rather than an already mated female in which sperm competition might occur. This difference in benefits (through the progeny fathered) probably explains the lack of increment of mating with an already mated female, especially as the female had been already mated by the same male; if the female was mated by another male, he could have gain by inseminating her and transferring at least some sperm, but the male does not gain any additional progeny by re-inseminating the same female. In males, opening their wings also increases patch residence time, although by a smaller factor than mating a virgin female (0.434 vs. 3.959). This is an expected result, since wings opening should equal the male perceiving the presence of a female.

Males discriminate the female quality and their tendency to stay on the patch increases only when mating with the high quality ones, i.e. the virgin ones, or performing behaviour linked to their presence such as wing opening. Similarly, females increase their patch time allocation when encountering healthy hosts, while encounters with parasitized hosts have no effect [43]; males and females *A. tabida* thus manage the information on resource quality the same way. In the parasitoid *Trichogramma euproctidis*, males also discriminate and prefer virgin females [57], but adjust their patch residence time based not on the number of mating with virgin females, but rather on the number of virgin females present on a patch [34]. In *A. tabida*, temporary exits are the only event decreasing the patch residence time. With the experimental set-up used in this study, exiting the patch also meant a drop in the perception of the former presence of females as no females were ever present outside the patch. It could then give the male an indication on the patch desertion, or on the patch surface. Alternatively, it could also mean that males that are about to leave the patch make more numerous temporary exits.

The efficiency of a given patch leaving rule of any organism depends on the ability to estimate patch quality, which in turns depends both on the ability to perceive patch quality [31] and the distribution of resource among patches [30]. The distribution of females among patches may explain the information use in decision mechanisms suggested in this study. Indeed, an incremental mechanism is adaptive when the resource is heterogeneously distributed and aggregated in a few patches in the environment, and no external cue is available to estimate patch quality [30], [31], [58], which is likely to be the case for both females in *A. tabida* [41], [43].

### Conclusion

This study extends the scope of the information used in the decisional mechanisms leading to patch departure. Until now, this type of decisional mechanism was suspected in a few numbers of organisms such as female parasitoids, bumblebees and human foraging for food and/or hosts [12], [22], [29]. This is the first study suggesting that males, as females, are able to manage the informative value of resource exploitation in accordance to an incremental proximal mechanism of patch departure. However, some studies are needed for a better understanding of the patch-leaving decision; testing the number of females, their quality (virgin or mated, by the same male or by another male, of different sizes and ages), the experience and physiological status of the male, and more would complete the information-use and patch-leaving decision rules understanding. Natural selection may favour emergence and evolution of mechanisms leading to efficient decision-making, such as patch departure, regardless of the biological basis of the mechanisms [16], [59], [60]. This adaptiveness of decision-making mechanisms may thus explain why organisms exploiting different types of resources (e.g. food, hosts or mates) with similar distribution and cues use the same proximal mechanism when facing the same environmental constraints.

## Supporting Information

Video S1 **A female** ***Asobara tabida*** **being glued on a hair during the patch preparation.** The female is anesthetised before the procedure, and the hair is unglued from the agar substrate once she awakens.
(WMV)
